# Host factor SMYD3 is recruited by Ebola virus nucleoprotein to facilitate viral mRNA transcription

**DOI:** 10.1080/22221751.2019.1662736

**Published:** 2019-09-13

**Authors:** Jingliang Chen, Zhangping He, Yaochang Yuan, Feng Huang, Baohong Luo, Jianhua Zhang, Ting Pan, Hui Zhang, Junsong Zhang

**Affiliations:** a Institute of Human Virology, Key Laboratory of Tropical Disease Control of Ministry of Education, Guangdong Engineering Research Center for Antimicrobial Agent and Immunotechnology, Zhongshan School of Medicine, Sun Yat-Sen University, Guangzhou, People’s Republic of China; b Department of Respiration, Affiliated Guangzhou Women and Children’s Hospital, Zhongshan School of Medicine, Sun Yat-Sen University, Guangzhou, People’s Republic of China; c CAS Key Laboratory for Pathogenic Microbiology, Institute of Microbiology, Chinese Academy of Sciences, Beijing, People’s Republic of China

**Keywords:** Ebola virus, NP, VP30, SMYD3, mRNA transcription

## Abstract

The polymerase complex of Ebola virus (EBOV) is the functional unit for transcription and replication of viral genome. Nucleoprotein (NP) is a multifunctional protein with high RNA binding affinity and recruits other viral proteins to form functional polymerase complex. In our study, we investigated host proteins associated with EBOV polymerase complex using NP as bait in a transcription and replication competent minigenome system by mass spectrometry analysis and identified SET and MYND domain-containing protein 3 (SMYD3) as a novel host protein which was required for the replication of EBOV. SMYD3 specifically interacted with NP and was recruited to EBOV inclusion bodies through NP. The depletion of SMYD3 dramatically suppressed EBOV mRNA production. A mimic of non-phosphorylated VP30, which is a transcription activator, could partially rescue the viral mRNA production downregulated by the depletion of SMYD3. In addition, SMYD3 promoted NP-VP30 interaction in a dose-dependent manner. These results revealed that SMYD3 was a novel host factor recruited by NP to supporting EBOV mRNA transcription through increasing the binding of VP30 to NP. Thus, our study provided a new understanding of mechanism underlying the transcription of EBOV genome, and a novel anti-EBOV drug design strategy by targeting SMYD3.

## Introduction

Ebola virus (EBOV) is an enveloped negative single-stranded RNA virus belonging to family *Filoviridae* in the order *Mononegavirales* [1]. Species *Zaire*, *Sudan*, *Reston*, *Tai Forest* and *Bundibugyo ebolavirus* constitute the genus *Ebolavirus*. Infection of EBOV can cause Ebola hemorrhagic fever (EHF) with high fatality rates in non-human primates and humans [2]. Causing over ten thousands of deaths in the West Africa outbreak between 2013 and 2015, EBOV emerges as one of the most lethal pathogens threatening public health worldwide [3].

The approximately 19-kb genome of EBOV contains 7 genes, encoding nucleoprotein (NP), virion protein of 35 kDa (VP35), VP40, glycoprotein (GP), VP30, VP24, and polymerase large protein (L) [4]. The transcription and replication of EBOV genome take place in the cytoplasm after virus entry [5]. The transcription of the viral genome generates individual primary mRNA species [6]. The viral mRNAs then utilize cellular translation machinery to produce viral proteins. Viral genomic RNA (vRNA) replicates to produce a full-length complementary RNA (cRNA), which serves as template to generate the vRNA in return. The transcription and replication competent polymerase complex of EBOV consists of NP, VP35, VP30 and L [5]. NP directly binds to VP35 and VP30 [7]. VP35 acts as a cofactor of RNA dependent RNA polymerase L, linking NP and L together. NP, VP35 and L are sufficient for viral genomic replication, while VP30 is indispensable for viral transcription [5,8]. The transcription activator function of VP30 is largely dependent on the phosphorylation states of two serine clusters (S29-S30-S31 and S42-S44-S46) in the N terminus [9,10]. Dephosphorylated VP30 has higher RNA binding affinity and stronger association with viral polymerase complex, and thus supports the viral transcription initiation and reinitiation, while highly phosphorylated VP30 favours viral replication [11–15]. NP recruits B56-PP2A phosphatase complex to dephosphorylate VP30 through a conserved LxxIxE motif to support viral mRNA transcription. The VP30 binding site is close to the B56-binding LxxIxE motif in NP, and both regions are required for VP30 dephosphorylation [16]. VP30 may also be dephosphorylated by PP1, however, the kinase(s) that phosphorylates VP30 is unknown [9,10].

NP is the most abundant viral protein in EBOV particles and in infected cells, and is essential for virus encapsidation and transcription and replication of viral genome. NP forms tube-like helical oligomers and binds to viral RNA *in vitro* and *in vivo*, which is the basic structure of tread-like virus particle [17,18]. Co-expression of NP, VP35 and VP24 is sufficient for the formation of typical EBOV nucleocapsid in cytoplasm [19,20]. EBOV forms inclusion bodies which are viral factories for virus replication in infected cells [21,22]. NP alone accumulates to form inclusions resembling inclusion bodies, indicating that NP functions as scaffold to recruit other proteins of viral polymerase complex for virus replication [7,23]. VP35 chaperons monomeric NP to prevent nascent NP binding to RNA, and mature NP oligomers undergo conformation change in order to bind RNA properly [24]. Optimized binding affinity between NP and VP30 is essential for viral RNA synthesis [5,25,26]. These observations suggest that NP plays key roles in the replication of EBOV.

In this study, we immunoprecipitated cellular proteins with NP in a transcription and replication competent EBOV minigenome system, and analysed the associated host proteins by mass spectrometry. Among these host factors, we focused on the role of SET and MYND domain-containing protein 3 (SMYD3) in the replication of EBOV. We found that SMYD3 specifically bound to NP, and the depletion of SMYD3 impaired the replication of EBOV, while overexpression of SMYD3 increased viral polymerase activity. SMYD3 mainly affected viral mRNA production, and a mimic of dephosphorylated VP30 partially rescued viral transcription when SMYD3 was depleted. We further found that SMYD3 promoted NP-VP30 interaction in a dose-dependent manner. Our results indicated that SMYD3 regulated the interaction between NP and the transcription activator VP30, and played a key role in viral mRNA transcription of EBOV.

## Materials and methods

### Cell culture and antibodies

Human embryonic kidney 293T (HEK293T) cells were maintained in Dulbecco’s modified Eagle’s medium (DMEM; Gibco) with 10% fetal calf serum (FBS; Gibco), 100 units/ml penicillin, and 100 μg/ml streptomycin (Gibco) at 37°C with 5% CO_2_.

The antibodies used in this study include: mouse monoclonal anti-HA (MBL), rabbit polyclonal anti-FLAG (MBL), rabbit polyclonal anti-GAPDH (Proteintech), rabbit polyclonal anti-SMYD3 (Proteintech), IRDye 680RD goat anti-rabbit (LI-COR), IRDye 800CW goat anti-mouse(LI-COR), Donkey Anti-Rabbit IgG H&L (Alexa Fluor® 594) (Abcam), Donkey Anti-Mouse IgG H&L (Alexa Fluor® 488) (Abcam).

### Plasmids

All plasmids encoding *Zaire ebolavirus* (strain Mayinga) viral proteins (pCAGGS NP, VP35, VP30 and L), pCAGGS T7 polymerase, EBOV minigenome (encoding *Renilla* luciferase), and tetracistronic minigenome (encoding *Renilla* luciferase, VP40, GP and VP24) have been described previously [5,27]. C-terminal tagged wild-type or mutant *Zaire ebolavirus* NP and VP30 were further cloned into pCAGGS with ClonExpress II (Vazyme) according to the manufacturer’s instructions. Furthermore, DNA sequences of *Reston ebolavirus* (strain Pennsylvania) NP (Re-NP) was chemically synthesized and C-terminal tagged wild-type and mutant Re-NP were constructed into pCAGGSS. cDNA of SMYD3, eIF3b, PABP1, G3BP1, TIAR-1 and B56α were obtained from HEK293T cell total mRNA and cloned into pCAGGS. All plasmid constructs were confirmed by DNA sequencing.

### Preparation of samples for mass spectrometry

HEK293T cells were seeded on 10 cm dish and transfected with 10 μg of pCAGGS-NP-HA, 750 ng of pCAGGS-VP35, 450 ng of pCAGGSVP30, 3 μg of pCAGGS-L, 1 μg of pCAGGS-T7 and 1 μg of EBOV minigenome. At 48 hours (h) after transfection, cells were collected and lysed with NP40 lysis buffer (10 mM Tris-HCl, pH7.4, 150 mM NaCl, 0.5% Nonidet P-40, 1% triton X-100, 2 mM EDTA, 10% glycerol) supplemented with protease inhibitor cocktail (Sigma Aldrich) on ice for 30 min. Lysates were clarified and rotated with anti-HA agarose beads for 4 h at 4°C. The beads were then washed for 4 times with 500 μl lysis buffer, boiled at 100°C with loading buffer supplemented with DTT and separated through 10% SDS-PAGE. The proteins were then visualized with ProteoSilver Plus Silver Stain Kit (Sigma Aldrich) according to the manufacturer’s instructions. The whole lane was cut into ten slices and prepared for liquid chromatography-tandem mass spectrometry (LC-MS/MS) analysis as described previously [28].

### siRNA transfection

HEK293T cells were transfected with 50 nM of negative control or SMYD3-speicfic siRNA (siSMYD3-1: 5’-GCAGAGTTGTCTTCAAACT-3’, siSMYD3-2: 5’-CTGAACGGCTTCCCGATAT-3’) (Ribobio) with Lipofectamine 2000 (Invitrogen) according to the manufacturer’s instructions. The cells were transfected with plasmids for EBOV minigenome assay or trVLP assay at 12 h after siRNA transfection.

### Western blotting

HEK293T Cells were collected and lysed with NP40 lysis buffer supplemented protease inhibitor cocktail (Sigma Aldrich) on ice for 30 min. Lysates were clarified, boiled at 100°C with loading buffer supplemented with DTT for 10 min and separated by SDS-PAGE. Proteins were transferred to nitrocellulose membranes (PALL). The membranes were then blocked with 5% non-fatty milk for 1 h at room temperature and incubated with primary antibodies overnight at 4°C. After 3 washed, membranes incubated with IRDye secondary antibodies (LI-COR) for 1 h at room temperature and scanned with the Odyssey infrared imaging system (LI-COR).

### Co-immunoprecipitation (co-IP) assay

HEK293T cells were seeded on 6 cm plates and transfected with plasmids as indicated. At 48 h after transfection, cells were collected and lysed with 500 μl NP40 lysis buffer supplemented with protease inhibitor cocktail (Sigma Aldrich) on ice for 30 min. Lysates were clarified by centrifugation and 50 μl of the lysates was taken as an input control. The remaining lysates were rotated with anti-HA agarose beads for 4 h at 4°C. The beads were then washed 4 times with 500 μl lysis buffer. Proteins were eluted with loading buffer supplemented with DTT at 100°C for 10 min. Input control and eluates were then analysed by western blotting.

### Immunofluorescence assay

HEK293T cells were seeded on chambered coverglass (ThermoFisher) and transfected as indicated. At 48 h after transfection, cells were washed twice with PBS and fixed with 4% poly-formaldehyde at room temperature for 10 min. The cells were then washed twice with PBS and incubated with 0.5% Triton X-100 at room temperature for 5 min, followed by PBS washing for 2 times and blocked with 5% bovine serum albumin PBS at room temperature for 30 min. Afterwards, the cells were incubated with primary antibodies at 4°C overnight. After 3 washed with 0.1% tween-20 PBS (PBS-T), the cells were incubated with secondary antibodies (Abcam) at room temperature for 45 min. Next, the cells were washed with 0.1% PBS-T for 2 times and incubate with DAPI at room temperature for 5 min, followed by washing with 0.1% PBST for 3 times. Images were obtained with LSM880 confocal microscopy (Zeiss).

### RNA isolation and quantitative real-time PCR (qRT-PCR)

HEK293T cells were transfected with siRNAs and plasmids for EBOV minigenome system. At 48 h after siRNA transfection, total RNA of cells was obtained using Trizol (life technologies) according to the manufacturer’s instructions. An amount of 1 μg total RNA was digested with RQ1 RNase-Free DNase (Promega) according to manufacturer’s instructions. 500 ng of the RNA was mixed with primer specific for mRNA (oligo dT), vRNA (5’-ATTGAAGATTCAACAACCCTAAAG-3’) or cRNA (5’-AATATGAGCCCAGACCTTTCG-3’) and then incubated at 65°C for 5 min and cooled to 4°C. The mixture was then reverse transcribed by PrimeScript RT reagent kit (Takara) according to the manufacturer’s instructions. Quantitative real-time PCR (qRT-PCR) was performed with SYBR Premix Ex Taq (TaKaRa) on a Bio-Rad CFX96 real time-PCR detection system (Bio-Rad) and PCR conditions are 95°C for 5 min, 40 cycles of 95°C for 10 s and 60°C for 30 s. Expression of EOBV RNAs was measured by RNA level of *Renilla* luciferase (forward: 5’-CGTCTAACCTCGCCCTTCTC-3’, reverse: 5’-AGGGCGAGAAAATGGTGCTT-3’) normalized to *Firefly* luciferase (forward: 5’-ACCGTCGTATTCGTGAGCAA-3’, reverse: 5’-GCTTTGGAAGCCCTGGTAGT-3’).

### EBOV minigenome assay

The assay has been described previously [5]. Briefly, 25 ng of pCAGGS-NP, 25 ng of pCAGGS-VP35, 15 ng of pCAGGSVP30, 200 ng of pCAGGS-L, 50 ng of EBOV minignome encoding a *Renilla* luciferase reporter gene, 50 ng of pCAGGS-T7 and 5ng of pGL4-cmv-*Firefly* luciferase were co-transfected into HEK293T cells for per well in 24-well plate with Lipofectamine 2000 (Invitrogen). In some experiments, VP30-6A was used as indicated. At 48 h after transfection, the cells were lysed and analysed with a dual-luciferase assay kit (Promega) according to the manufacturer’s instructions. Replication of EBOV was measured by *Renilla* luciferase activity normalized to *Firefly* luciferase values.

### EBOV trVLP assay

The assay was performed previously [29]. In brief, 125 ng of pCAGGS-NP, 125 ng of pCAGGS-VP35, 75 ng of pCAGGSVP30, 1 μg of pCAGGS-L, 250 ng of an EBOV tetracistronic minigenome (encoding a *Renilla* luciferase reporter gene, VP40, GP, and VP24), and 250 ng of pCAGGS-T7 were co-transfected into HEK293T cells (producer cells) for per well in 6-well plate with Lipofectamine 2000 (Invitrogen). After 12 h, cell culture medium was discarded and replaced with fresh medium. At 72 h after transfection, supernatants containing Ebola trVLPs were collected, filtered through a 0.45 μm pore-size filter (Pall) and stored at −80°C until use. For infection of the trVLPs, HEK293T cells (target cells) were plated in 24-well plate and transfected with 25 ng of pCAGGS-NP, 25 ng of pCAGGS-VP35, 25 ng of pCAGGSVP30, 200 ng of pCAGGS-L, 50 ng of pCAGGS-Tim-1 and 5 ng of pGL4-cmv-*Firefly* luciferase. At 24 h after transfection, medium was discarded and infected with trVLPs. At 12 h after infection, medium was discarded and replaced with fresh medium. At 48 h after infection, target cells were lysed and analysed with a dual-luciferase assay kit (Promega) according to the manufacturer’s instructions.

### Statistical analysis

A two-tailed Student *t-*test was used to determine the significance of statistical data. Data were considered significant at *P < 0.05, **P < 0.01 and ***P < 0.001.

## Results

### Identification of SMYD3 as a host factor facilitating the replication of EBOV

**Figure 1.**
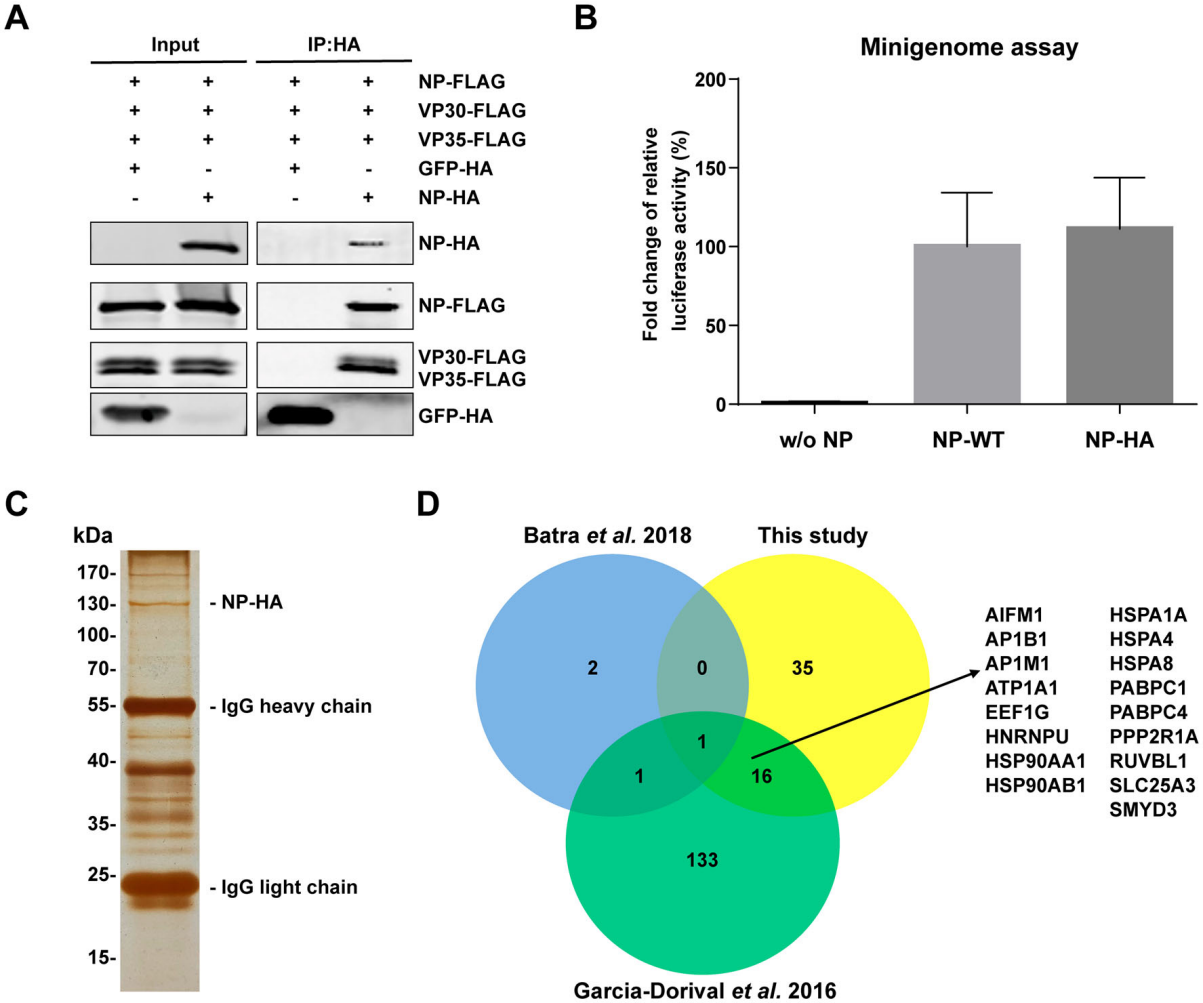
Identification of host proteins associated with transcription and replication competent polymerase complex of EBOV. (A) C-terminal HA-tagged NP interacted with other viral proteins of EBOV polymerase complex. Plasmids encoding GFP-HA (negative control) or NP-HA were co-transfected separately with NP-FLAG, VP35-FLAG and VP30-FLAG into HEK293T cells. At 48 h post-transfection (p.t.), cells were collected, followed by anti-HA co-IP assay and western blotting. Representative results of 3 independent experiments are shown. (B) NP-HA rescued EBOV replication in the minigenome system. Plasmids of EBOV viral proteins (VP35, VP30, L, and empty vector, non-tagged NP or NP-HA), T7 polymerase, EBOV minigenome and pGL4 were co-transfected into HEK293T cells. At 48 h p.t., cells were collected, followed by dual-luciferase assay. The ratio of *Renilla* to *Firefly* luciferase activity of lysates from cells transfected with non-tagged NP was set to 100%. The mean and SEM from one representative experiment (n = 3) of 3 independent experiments are indicated. (C) Immunoprecipitation of host proteins associated with EBOV polymerase complex using NP-HA as bait. Plasmids expressing NP-HA, VP35, VP30, L, T7 polymerase and EBOV minigenome were co-transfected into HEK293T cells. At 48 h p.t., cells were lysed, followed by anti-HA immunoprecipitation. Eluate was separated by SDS-PAGE and visualized by silver staining. (D) Venn diagram showing the overlap of candidate host proteins interacting with NP between this study and two previous studies. Seventeen proteins that were identified both in our study and the study by Garcia-Dorival et al. are listed [30].

To identify host factors associated with EBOV NP in a physiological condition, we utilized immunoprepitation (IP) with NP in a transcription and replication competent polymerase complex system. We firstly tested interactions between a C-terminal HA-tagged NP (NP-HA) and other viral proteins. As expected, NP-HA could interact with VP35 and VP30, and form homo-oligomers at the same time (Figure 1(A)). We took advantage of an EBOV minigenome system to examine the biological function of NP-HA in replication of EBOV [5]. Cells were transfected with plasmids encoding EBOV NP, VP35, VP30, L, T7 polymerase, a T7 promoter-driven EBOV monocistronic minigenome (encodes a single gene, *Renilla* luciferase, flanked by viral genome 3’ leader and 5’ trailer sequences), and a control reporter encoding *Firefly* luciferase in this system. The relative activity of *Renilla* to *Firefly* luciferase reflects the transcription and replication of EBOV genome. In the minigenome system, we found that NP-HA had similar activity in supporting polymerase function compared with untagged NP (Figure 1(B)). These results indicated that NP-HA was as competent as untagged NP in supporting the replication of EBOV. We then purified host proteins with NP-HA in this validated EBOV minigenome system. Cells were transfected plasmids encoding NP-HA, other viral proteins, T7 polymerase, and EBOV minigenome, and then lysed for anti-HA immunoprecipitation at 48 h post-transfection (Figure 1(C)). The associated host proteins were analysed with mass spectrometry, and we obtained 52 proteins that potentially interacted with NP (Supplementary Table 1). Comparing our results to two previous studies which also aimed to identify NP-associating host factors, we found that AP1M1, a subunit of adaptor protein complex-1, was the only protein shared by all the three results (Figure 1(D)) [30,31]. AP1M1 facilitates dengue virus (DENV) RNA replication but is not involved in virus binding and internalization [32]. Another sixteen proteins were also identified as interactors of NP by co-IP using NP-GFP as bait (Figure 1(D)) [30]. Among these proteins, HSP90 and HSP70 interact with NP, and HSP70 is require for replication of EBOV by promoting stability of NP [30]. Two poly-(A)-binding protein, PABPC1 and PABPC4, were in the shared list. PABPC1 plays positive role in DENV replication and both proteins may be involved in the replication of EBOV [33]. Interestingly, there were 5 RNA helicases uniquely identified in our study, among which DDX21, DDX3X, and DHX9 play roles in RNA synthesis of several viruses [34–38]. We noticed that SET and MYND domain-containing protein 3 (SMYD3) was a potential host factor associating with NP. SMYD3 belongs to the SET/MYND domain protein family consisting of SMYD1-5. SMYD3 mediates the methylation of histone H3 and H4, and several none histone proteins [39–43], and interacts with Pol II, BRD4 and PC4, playing important roles in mRNA transcription [43–45]. Considering the positive role of SMYD3 in cellular RNA synthesis, we decided to further study its function in the replication of EBOV.

**Figure 2.**
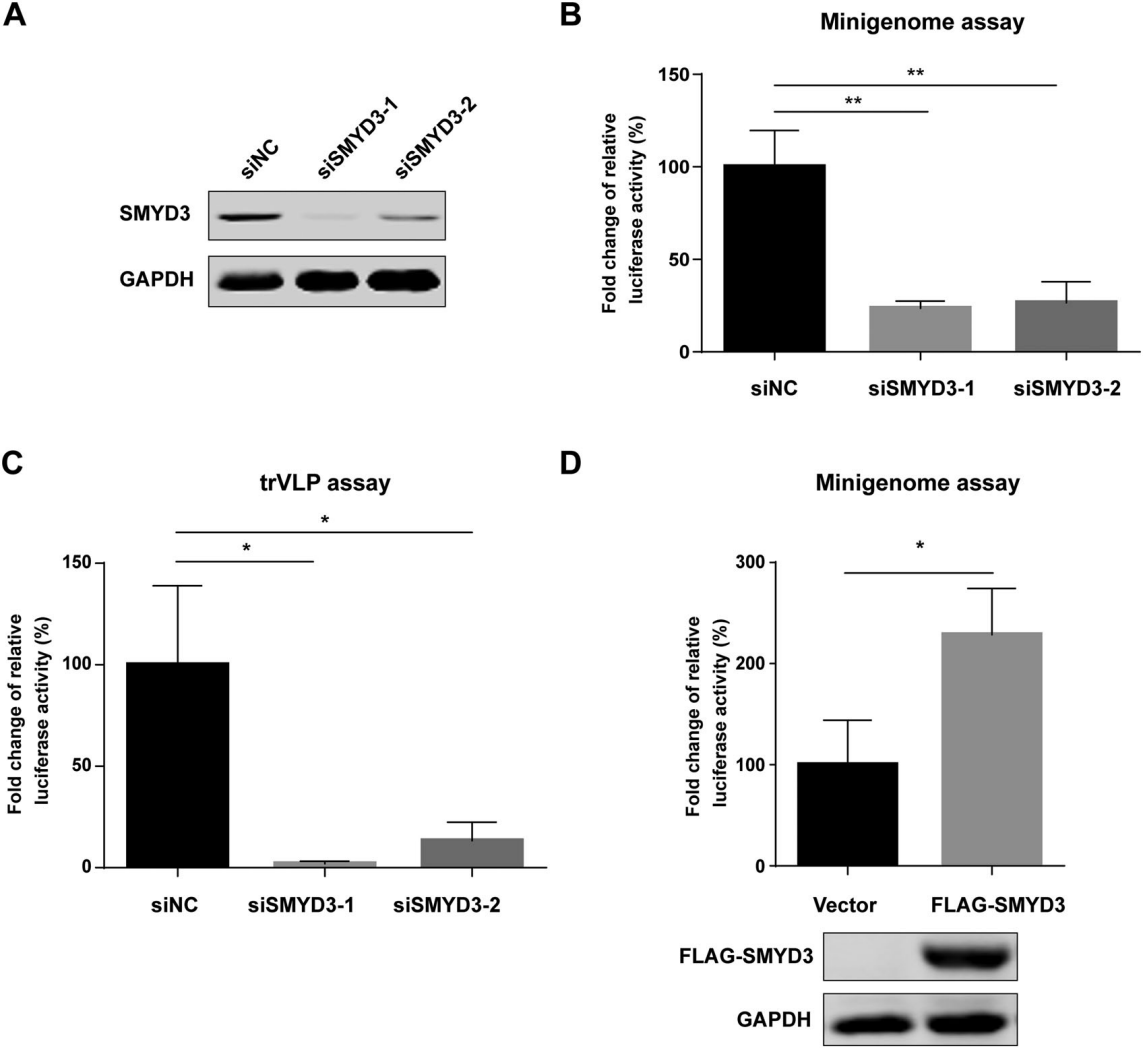
SMYD3 was required for the replication of EBOV. (A) Depletion of SMYD3 protein expression by siRNA. HEK293T cells were transfected with negative control siRNA (siNC) or two SMYD3 specific siRNA, siSMDY3-1 and siSMYD3-2. Cells were collected 48 h p.t., followed by western blotting. Representative results of at least 3 independent experiments are shown. (B) Depletion of SMYD3 impaired the replication of EBOV in minigenome system. HEK293T cells were transfected with siNC, siSMYD3-1 or siSMYD3-2. Twelve hours later, cells were transfected plasmids for EBOV minigenome system. Cells were collected 48 h after siRNA transfection, followed by dual-luciferase assay. The ratio of *Renilla* to *Firefly* luciferase activity of lysates from cells treated with siNC was set to 100%. The mean and SEM from one representative experiment (n = 3) of 3 independent experiments are indicated. (C) Depletion of SMYD3 impaired the replication of EBOV in trVLP system. HEK293T cells were transfected with siNC, siSMYD3-1, or siSMYD3-2. Twelve hours later, cells were transfected with plasmids encoding NP, VP35, VP30, L and Tim-1. At 24 h p.t., medium was discarded and cells were infected with Ebola trVLPs. At 12 h post-infection (p.i.), supernatants were discarded and replaced with fresh medium. At 48 h p.i., cells were collected, followed by dual-luciferase assay. The ratio of *Renilla* to *Firefly* luciferase activity of lysates from cells treated with siNC was set to 100%. The mean and SEM from one representative experiment (n = 3) of 3 independent experiments are indicated. (D) Over-expression of SMYD3 promoted the replication of EBOV. HEK293T cells were transfected with empty vector or plasmid encoding FLAG-SMYD3. Twenty-four hours later, cells were transfected with plasmids for EBOV minigenome system. Cells were collected 48 h after the second transfection, followed by dual-luciferase assay and western blotting. The ratio of *Renilla* to *Firefly* luciferase activity of lysates from cells transfected with empty vector was set to 100%. The mean and SEM from one representative experiment (n = 3) of 3 independent experiments are indicated. *P < 0.05*,* **P < 0.01 (student’s *t*-test).

To examine the effect of SMYD3 on viral transcription and replication, we tested polymerase activity after knockdown of SMYD3 using SMYD3-specific siRNAs. The depletion efficiency of SMYD3 by two siRNA was confirmed by western blotting (Figure 2(A)). After depletion of SMYD3, the replication of EBOV was significantly impaired in minigenome system (Figure 2(B)). We next took advantage of a transcription and replication competent virus-like particle (trVLP) system, which could better simulate life cycle of wild-type virus, to investigate the role of SMYD3 in the replication of EBOV [29]. In this system, the modified EBOV genome contains *Renilla* luciferase reporter gene, VP40, GP, and VP24. This tetracistronic genome allows self-modulated expression of VP40 and VP24 to avoid their negative effect on viral transcription and replication when they are provided *in tans*, and thus produces robust viral particles in the presence of NP, VP35 VP30 and L in producer cells [46,47]. Target cells were infected with the trVLPs and viral transcription and replication were monitored by luciferase reporter assay. Replication of EBOV was significantly decreased in target cells treated with SMYD3 specific siRNAs compared to those treated with negative control siRNA after infection of trVLPs (Figure 2(C)). In addition, the replication of EBOV was enhanced by overexpression of SMYD3 (Figure 2(D)). Collectively, our data showed that host factor SMYD3 was associated with EBOV polymerase complex, and required for the efficient replication of EBOV.

### SMYD3 specifically interacted with EBOV NP

**Figure 3.**
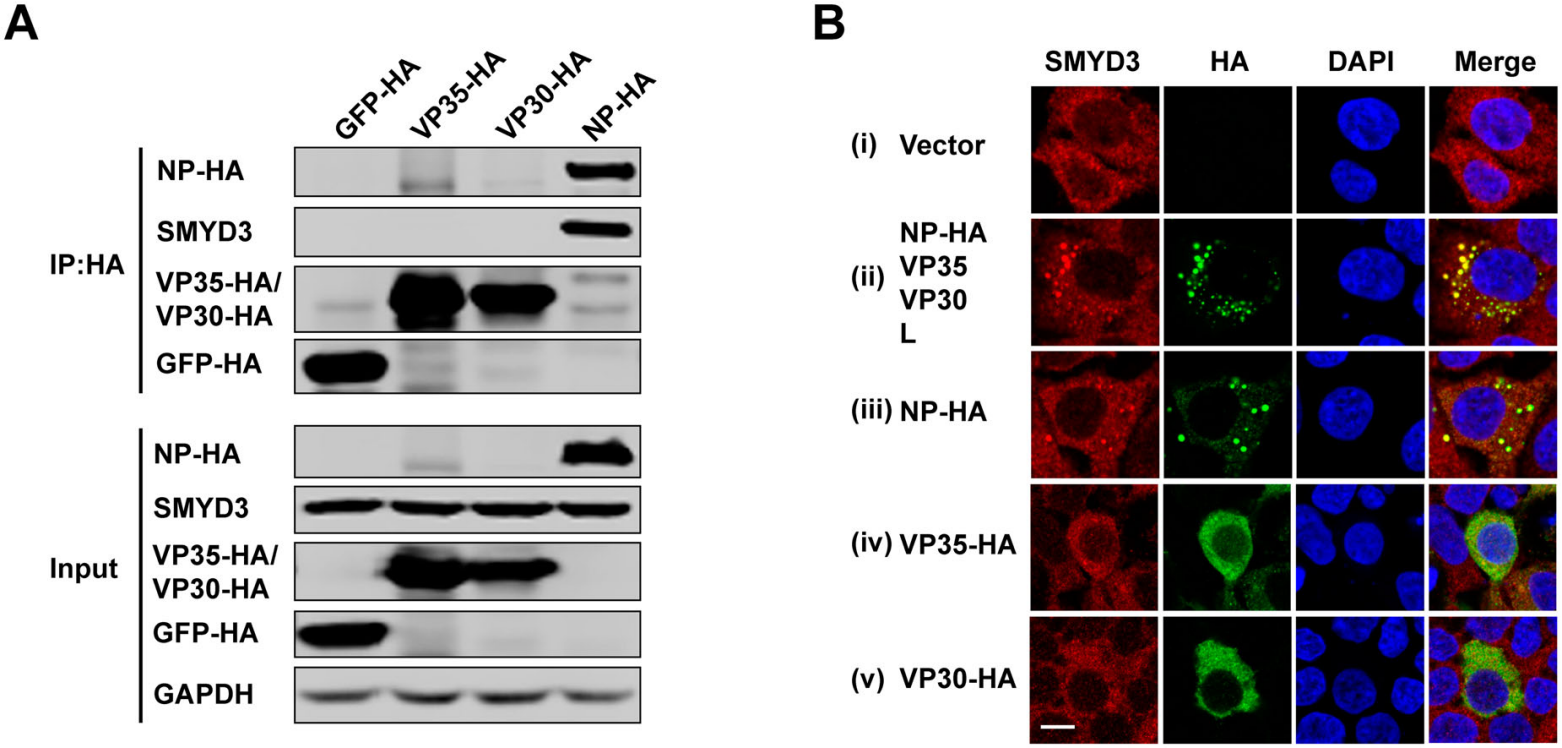
SMYD3 specifically interacted with EBOV NP. (A) Endogenous SMYD3 was co-purified with NP. Plasmids encoding GFP-HA, VP35-HA, VP30-HA and NP-HA were separately transfected into HEK293T cells. Cells were collected 48 h p.t., followed by anti-HA co-IP assay and western blotting. Representative results of 3 independent experiments are shown. (B) SMYD3 was recruited to EBOV inclusion bodies by NP. HEK293T cells were transfected with empty vector (panel i) or plasmids encoding NP-HA, VP35, VP30 and L (panel ii), NP-HA (panel iii), VP35-HA (panel iv) or VP30-HA (panel v). At 48 h p.t., cells were fixed with 4% poly-formaldehyde, followed by Immunofluorescence assay (IFA). Scale bar, 10 μm.

To examine the interaction(s) between SMYD3 and viral proteins of EBOV polymerase complex, HA-tagged NP, VP35, VP30 and negative control GFP were transfected separately into HEK293T cells and anti-HA co-immunoprecipitation (co-IP) was performed using anti-HA agarose beads. We found that endogenous SMYD3 could only be co-purified with NP (Figure 3(A)), suggesting that SMYD3 was associated with EBOV polymerase through interaction with NP. During infection, EBOV forms typical inclusion bodies in cytoplasm, which consist of viral proteins and are sites for virus replication [21,22]. As SMYD3 was required for replication of EBOV, we next investigated whether SMYD3 was recruited into viral inclusion bodies. Endogenous SMYD3 distributed homogenously in cytoplasm in the absence of viral proteins (Figure 3(B), panel i). When EBOV NP, VP35, VP30, and L were co-transfected into cells, inclusion bodies were formed and endogenous SMYD3 was recruited to sites of inclusions (Figure 3(C), panel ii). Transfection of NP induced inclusions, and SMYD3 was relocated to these inclusions (Figure 3(C), panel iii), which was consistent with the co-IP result. In the parallel experiments, when expressed alone, VP35 or VP30 showed homogenous distribution in the cytoplasm, and no obvious colocalization was found between SMYD3 and these two viral proteins (Figure 3(C), panel iv and v).

During EBOV infection, several stress granule (SG) proteins colocalized with inclusion bodies, including eIF4G, eIF3b, PABP1, G3BP1 and TIAR-1 [48,49]. There may be possibility that these proteins mediated the localization of SMYD3 to NP-induced inclusions. We firstly examined whether these SG proteins could colocalize with NP. As eIF4G could not be recruited to NP inclusions, we investigated the other four SG proteins [48]. HA-tagged eIF3b, PABP1, G3BP1 or TIAR-1 was separately co-transfected with NP-FLAG and their localizations were examined. None of these SG proteins was colocalized with NP-induced inclusions (Supplementary Fig. 1). Thus, recruitment of SMYD3 into NP-induced inclusions was not mediated by eIF4G, eIF3b, PABP1, G3BP1 or TIAR-1.

In summary, we found that SMYD3 specifically bound to EBOV NP and was recruited to EBOV inclusion bodies through the interaction with NP.

### Analyses of regions responsible for the interaction between SMYD3 and NP

**Figure 4.**
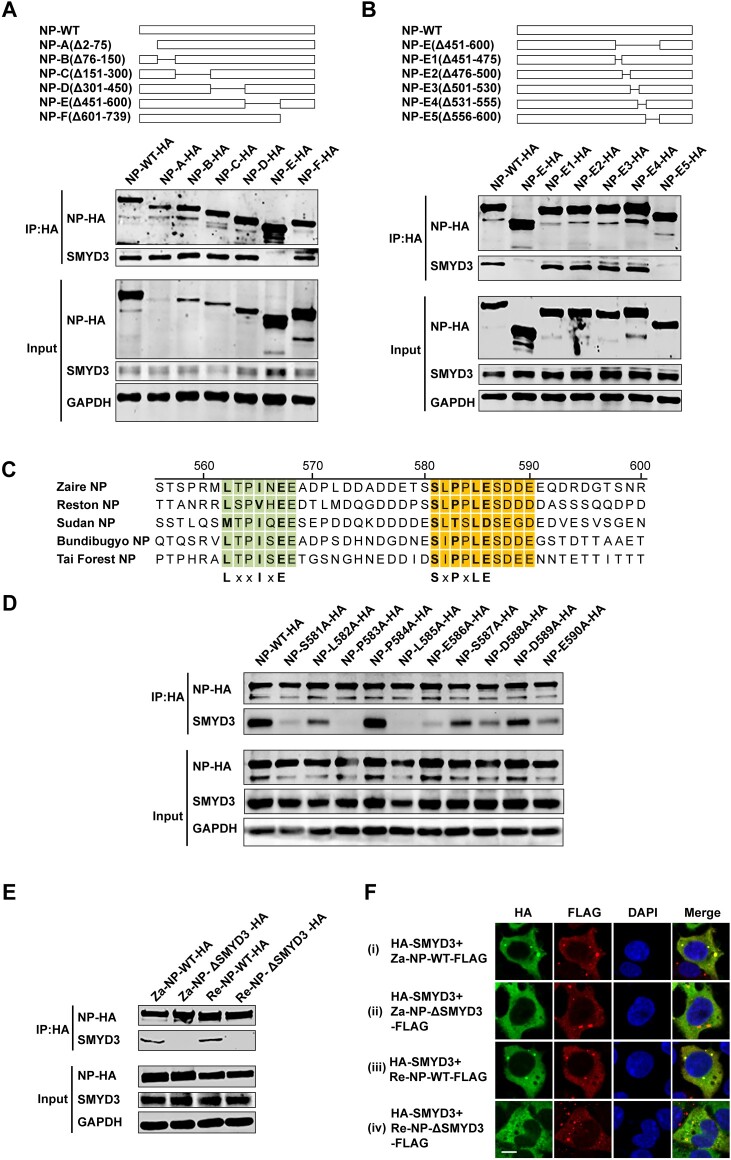
Mapping binding sites of NP interacting with SMYD3. (A) NP mutant with AA residues 450-600 truncated could not co-purify SMYD3. Schematic of full-length NP and 5 primary truncated mutants (top panel). Wild-type NP and mutants were separately transfected to HEK293T cells. Cells were collected 48 h p.t., followed by anti-HA co-IP assay and western blotting (bottom panel). Representative results of at least 3 independent experiments are shown. (B) Amino acid residues 556-600 of NP were essential for the interaction between NP and SMYD3. Illustration of full-length NP and deletion mutants (top panel). HEK293T cells were transfected with wild-type NP or mutants as indicated. Cells were collected 48 h p.t., followed by anti-HA co-IP assay and western blotting (bottom panel). Representative results of at least 3 independent experiments are shown. (C) Alignment of NP amino acid sequences within residues 556-600 among five EBOV species. The LxxIxE and SxPxLE motif are indicated below. (D) NP bound to SMYD3 through an SxPxLE motif. HEK293T cells were transfected with wild-type NP or single point mutants as indicated. Cells were collected 48 h p.t., followed by anti-HA co-IP assay and western blotting. Representative results of 3 independent experiments are shown. (E) The interaction between SMYD3 and NP was conserved in different EBOV species. HEK293T cells were transfected with Za-NP-WT, Za-NP-ΔSMYD3, Re-NP-WT or Re-NP-ΔSMYD3 as indicated. Cells were collected 48 h p.t., followed by anti-HA co-IP assay and western blotting. Representative results of at least 3 independent experiments are shown. (F) NP-ΔSMYD3 could not recruit SMYD3 into its inclusions. C-terminal FLAG-tagged Za-NP-WT, Za-NP-ΔSMYD3, Re-NP-WT or Re-NP-ΔSMYD3 was transfected separately with HA-SMYD3 into HEK293T cells. Cells were fixed 48 h p.t., followed by IFA. Scale bar, 10 μm.

To map the region(s) of NP interacting with SMYD3, six truncated species of NP were generated, including NP-A(Δ2-75), NP-B(Δ76-150), NP-C(Δ151-300), NP-D(Δ301-450), NP-E(Δ451-600) and NP-F(Δ601-739) (Figure 4(A) top panel). In co-IP assay, NP-E(Δ451-600) was found to be the only mutant that lost the binding to endogenous SMYD3 (Figure 4(A) bottom panel). Five mutants with deletions within amino acid (AA) 451-600 of NP were further constructed to search for regions binding to SMYD3 (Figure 4(B) top panel), among which only NP-E5 (Δ556-600) could not bind to SMYD3 (Figure 4(B) bottom panel). Interestingly, multiple sequence alignment of NP AA of five species of EBOV showed that there were two relatively conserved regions within AA 556-660, one is the LxxIxE motif which is responsible for interaction of NP with B56, another is an SLPPLESDDE sequence (Figure 4(C)) [16]. We speculated that later region was responsible for NP-SMYD3 interaction. To test this hypothesis, we constructed NP mutants with single point mutation within the SLPPLEDSDDE sequence and investigate their binding to SMYD3. The co-IP results revealed that most of these mutants exerted impaired function in binding to SMYD3, especially NP-S581A, NP-P583A, NP-L585A and NP-E586A (Figure 4(D)), suggesting that an SxPxLE motif in NP was responsible for interaction with SMYD3. To explore whether the interaction between NP and SMYD3 was conserved among different EBOV species, we generated plasmids encoding wild-type Reston virus NP (Re-NP-WT) and Reston virus NP-L585A (Re-NP-ΔSMYD3). In co-IP experiments, Re-NP-WT co-purified SMYD3 as efficiently as wild-type Zaire virus NP (Za-NP-WT), whereas Re-NP-ΔSMYD3 could not bind to SMYD3 (Figure 4(E)). Re-NP-WT recruited SMYD3 to inclusions as Za-NP-WT did, while Re-NP-ΔSMYD3 and Zaire NP-L585A (Za-NP-ΔSMYD3) did not (Figure 4(F)). Taken together, our studies demonstrated that EBOV NP bound to SMYD3 through a relatively conserved SxPxLE motif and the NP-SMYD3 interaction was conserved among EBOV species.

**Figure 5.**
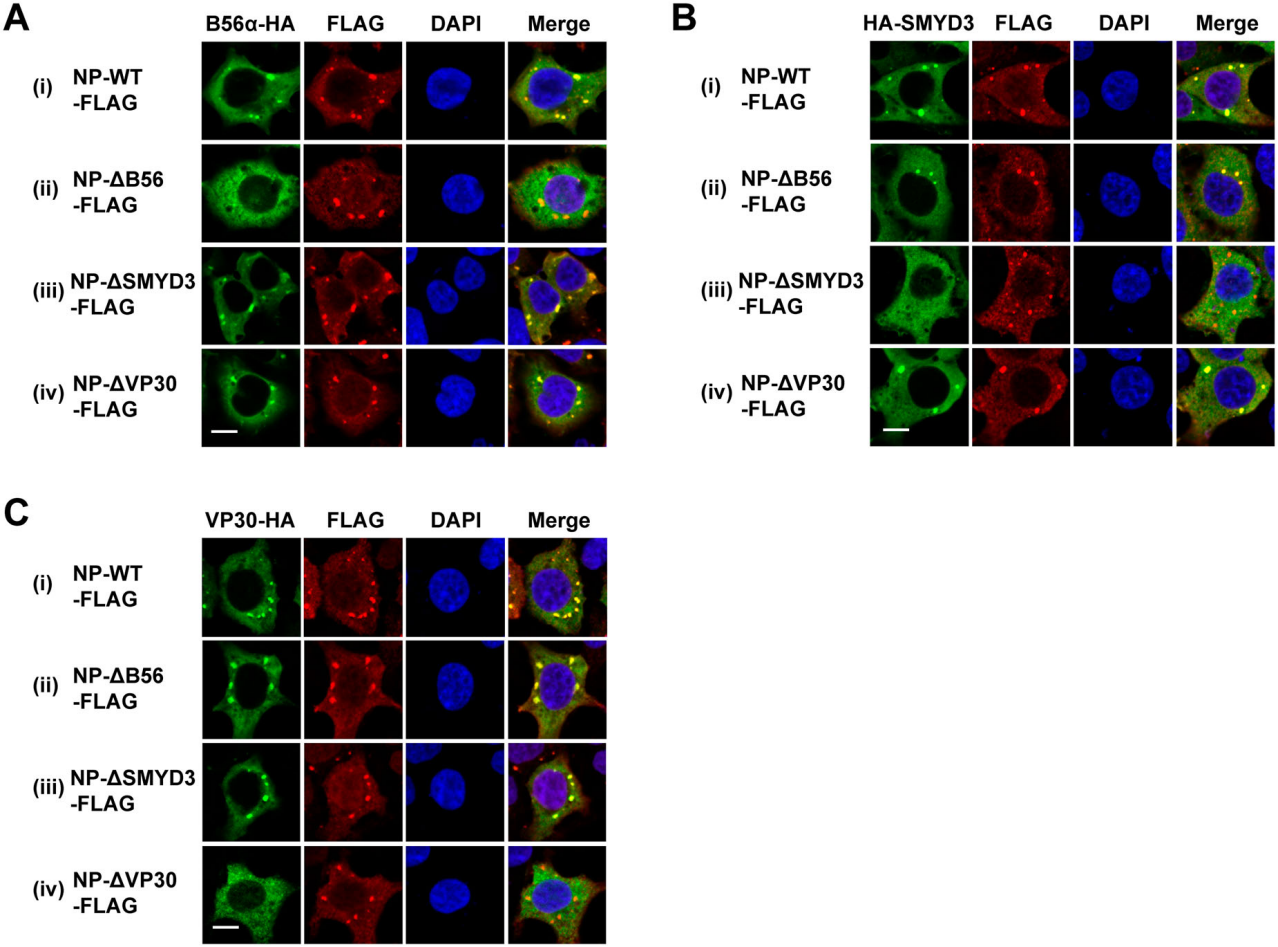
Colocalization of B56α, SMYD3 and VP30 with NP-WT-, NP-ΔB56-, NP-ΔSMYD3- and NP-ΔVP30-induced inclusions. B56α-HA (A), HA-SMYD3 (B), or VP30-HA (C) were co-transfected with C-terminal FLAG-tagged NP-WT, NP-Δ56, NP-ΔSMYD3 or NP-ΔVP30 into HEK293T cells. Cells were fixed 48 h p.t., followed by IFA. Scale bar, 10 μm.

The regions of NP responsible for interaction with B56, SMYD3 and VP30 are AA 562-567, 581-590 and 603-612, respectively, which are of close proximity [16,25,26]. We sought to investigate whether the failure of colocalization with NP of one protein influences that of the other two proteins. To this end, NP-WT, NP-L562A/I565A/E567A (NP-ΔB56), NP-ΔSMYD3 or NP-603-606A/610-612A (NP-ΔVP30) was separately co-transfected with B56α, SMYD3 or VP30, and their localizations were examined. As expected, no colocalization between NP-ΔB56 and B56α or NP-ΔVP30 and VP30 was observed (Figure 5(A) panel ii and (C) panel iv). However, NP-ΔB56, NP-ΔSMYD3 and NP-ΔVP30 could recruit SMYD3 and VP30 (Figure 5(A) panel iii and iv), B56α and VP30 (Figure 5(B) panel ii and iv), and B56α and SMYD3 (Figure 5(C) panel ii and iii) to inclusions, respectively. In summary, loss-of-function mutations of B56, SMYD3 or VP30 binding motif did not prevent NP recruiting the other two proteins into its inclusions.

**Figure 6.**
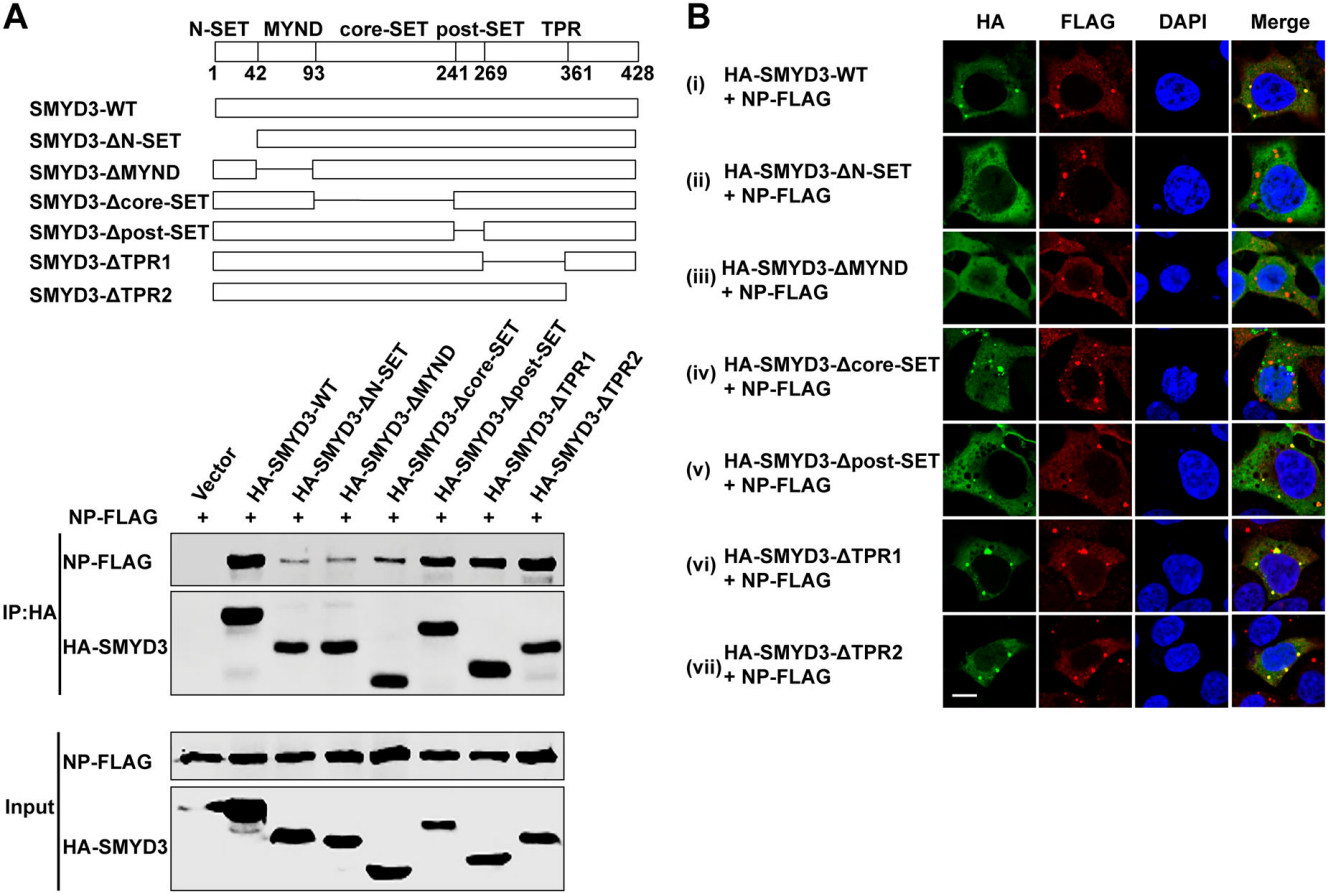
Mapping regions of SMYD3 binding to NP. (A) AA 2-240 of SMYD3 mediated interaction with NP. Illustration of full-length SMYD3 and truncated mutants (top panel). HA-tagged wild-type SMYD3 and truncated mutants were separately transfected with NP-FLAG into HEK293T cells. Cells were collected 48 h p.t., followed by anti-HA co-IP assay and western blotting (bottom panel). Representative results of at least 3 independent experiments are shown. (B) AA 2-240 of SMYD3 were responsible for relocation to NP-induced inclusions. N-terminal HA-tagged wild-type SMYD3 and truncated mutants were separately transfected with NP-FLAG into HEK293T cells. Cells were fixed 48 h p.t., followed by IFA. Scale bar, 10 μm.

SMYD3 contains five domains [50,51]. A SET domain, a conserved catalysis region for lysine methylation, is split into an N-SET and a core-SET domain by a MYND domain, which is a zinc finger motif contribute to protein–protein and protein–DNA interaction. The SET domain is followed by a post-SET domain which supports cofactor and substrate binding, and a tetratricopeptide repeats-like (TPR) structure. We generated internal truncated SMYD3 to map region(s) binding to NP according to its crystal structure (Figure 6(A) top panel). In co-IP experiments, although none of these mutants completely lost the binding to NP, SMYD3 without N-SET, MYND or core-SET domain showed impaired function in co-purifying NP (Figure 6(A) bottom panel). In immunofluorescence assay, SMYD3-ΔN-SET and SMYD3-ΔMYND could not colocalize with NP-induced inclusions (Figure 6(B) panel ii and iii). Interestingly, SMYD3-Δcore-SET formed aggregates in the cytoplasm, however, these aggregates could not colocalize with NP inclusions (Figure 6(B) panel iv). While other mutants were still redirected to NP-accumulating sites (Figure 6(B) panel v-vii). All together these results showed that the N-SET, MYND and core-SET domains of SMYD3 were important for the interaction with EBOV NP.

### SMYD3 promoted the replication of EBOV through facilitating viral mRNA production

**Figure 7.**
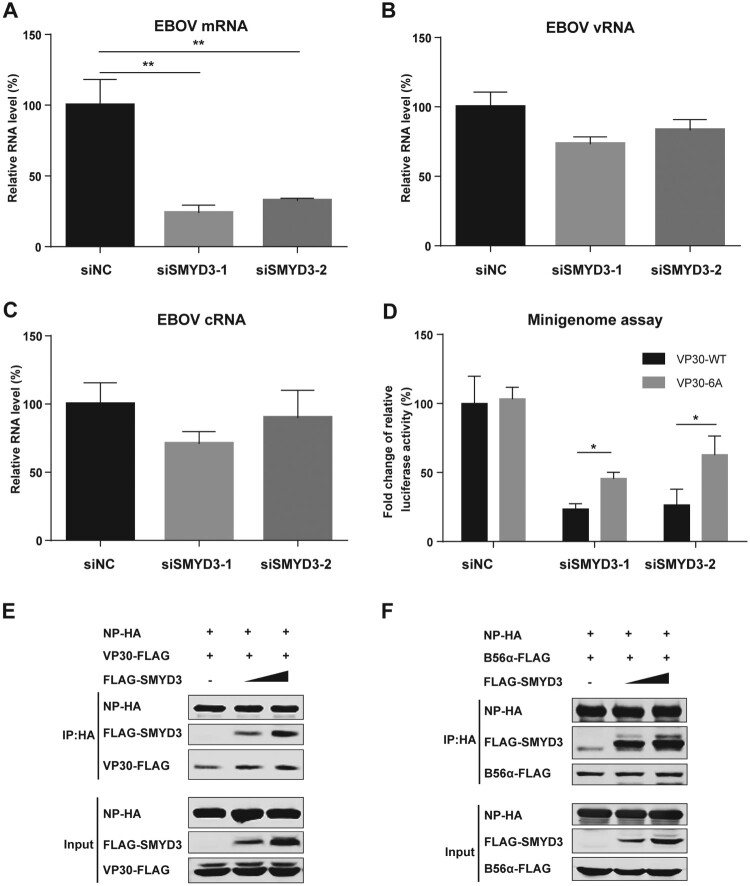
SMYD3 supported EBOV replication by facilitating mRNA production. (A-C) Depletion of SMYD3 down-regulated mRNA production of EBOV. HEK293T cells were transfected with siNC, siSMYD3-1 or siSMYD3-2. At 12 h p.t., plasmids for EBOV minigenome system were transfected. At 48 p.t. of siRNA, cells were collected and RNAs were extracted and analysed by qRT-PCR to measure expression levels of EBOV mRNA (A), vRNA (B), and cRNA (C). Viral mRNA, vRNA and cRNA were reverse transcribed by specific primer. qRT-PCR were performed to measure the relative RNA levels of *Renilla* to *Firefly* luciferase. The relative RNA level of cells treated with siNC were set to 100%. The mean and SD from one representative experiment (n = 3) of 3 independent experiments are indicated. (D) VP30-6A rescued replication of EBOV after depletion of SMYD3. HEK293T cells were transfected with siNC, siSMYD3-1 or siSMYD3-2. Twelve hours later, cells were transfected with plasmids of EOBV proteins (NP, VP35, L, and VP30-WT or VP30-6A), T7 polymerase and EBOV minigenome. At 48 h after transfection of siRNA, Cells were collected, followed by dual-luciferase assay. The ratio of *Renilla* to *Firefly* luciferase activity of lysates from cells transfected with siNC and VP30-WT was set to 100%. The mean and SEM from one representative experiment (n = 3) of 3 independent experiments are indicated. (E) SMYD3 promoted NP-VP30 interaction in a dose-dependent manner. HEK293T cells were transfected with NP-HA, VP30-FLAG and increasing amounts of FLAG-SMYD3. Empty vector was used to balance the total amounts of plasmids transfected. At 48 h p.t., cells were collected, followed by anti-HA co-IP assay and western blotting. Representative results of 3 independent experiments are shown. (F) SMYD3 had no effect on NP-B56 interaction. NP-HA, B56α-FLAG and increasing amounts of FLAG-SMYD3 were co-transfected into HEK293T cells. Empty vector was used to balance the total amounts of plasmids transfected. At 48 h p.t., cells were collected, followed by anti-HA co-IP assay and western blotting. Representative results of 3 independent experiments are shown. *P < 0.05*,* **P < 0.01 (student’s *t*-test).

We next sought to explore the mechanism of SMYD3 in supporting the replication of EBOV. SMYD3, B56 and VP30 binding sites in NP are close to each other [16,25,26]. As VP30 and PP2A-B56 phosphatase play important role in EBOV mRNA transcription, we hypothesize that SMYD3 might be involved in the process [5,8,16]. After depletion of SMYD3, we found that viral mRNA was significantly decreased, while vRNA and cRNA only showed modest changes (Figure 7(A-C)). EBOV mRNA transcription is activated by dephosphorylated VP30. Dephosphorylation of VP30 by PP1 and PP2A promotes its activity in supporting viral transcription [10,16]. Okadaic acid could inhibit the catalytic activity of PP1 and PP2A, and thus inhibited EBOV viral transcription [9,12]. NP mutant which did not bind to PP2A-B56 phosphatase could not support viral mRNA transcription. VP30-6A with all the six serines of S29-S30-S31 and S42-S44-S46 mutated to alanine, mimicking non-phosphorylated VP30, supported viral mRNA transcription in the presence of okadaic acid or when NP lost PP2A-B56 binding ability. As SMYD3 was important for mRNA production, we hypothesized that VP30-6A could rescue replication of EBOV when SMYD3 was depleted. When transfected in the replacement of wild-type VP30 (VP30-WT) in the minigenome system, VP30-6A could partially rescue the impaired replication of EBOV induced by SMYD3-specific siRNA treatment, while it showed similar activity as VP30-WT in cells treated with negative control siRNA (Figure 7(D)). These results suggested that SMYD3 was involved in VP30-dependent mRNA transcription. We speculated that SMYD3 might strengthen the binding of B56 or VP30 to NP. We transfected increasing doses of SMYD3 and examined the amounts of B56α or VP30 co-purified with NP. Results showed the amounts of VP30 pulled-down by NP were increased in a SMYD3 dose-dependent manner (Figure 7(E)), while those of B56α co-purified with NP were not affected (Figure 7(F)), suggesting SMYD3 promoting the interaction of NP and VP30, but not that of NP and B56α.

In conclusion, our data showed that depletion of SMYD3 inhibited EBOV mRNA transcription, which could be partially rescued by VP30-6A, and SMYD3 supported vial mRNA transcription through strengthening NP-VP30 interaction.

## Discussion

EBOV NP binds strongly to viral RNA and acts as scaffold to recruit other viral protein for transcription and replication of virus genome [7,23]. However, the host factors that interact with NP are largely unknown. In this study, we identified host proteins associated with EBOV polymerase complex using NP as bait by immunoprecipitation and LC-MS/MS analysis. We found that SMYD3 was a unique host factor associated with EBOV polymerase complex. Depletion of SMYD3 impaired replication of EBOV in both minigenome system and trVLP system, indicating its important role in EBOV life cycle.

Co-IP results showed that SMYD3 specifically bound to NP. EBOV forms aggregated inclusion bodies in cytoplasm of host cells. SMYD3, which homogenously distributed in the cytoplasm in cells under normal condition, was relocated to inclusion bodies formed by EBOV polymerase complex. Consistent with the co-IP results, SMYD3 was redirected to NP-induced inclusions when NP is the only viral protein provided. Studies on mapping regions of NP binding to SMYD3 showed that a SxPxLE motif was essential for the interaction. A single AA mutation of residue 585 from leucine to alanine in NP abolishes the interaction between NP and SMYD3 (NP-ΔSMYD3). NP-ΔSMYD3 induced inclusions in cytoplasm as wild-type NP did, however, it lost the ability to relocate SMYD3 into the inclusions. The SxPxLE motif is relatively conserved in between different EBOV species. Indeed, Reston virus NP bound to SMYD3 and relocated SMYD3 into inclusions, while Re-NP-ΔSMYD3 lost these functions. Interestingly, the binding site of SMYD3 in NP located in between those of B56 and VP30 [16,25,26]. Our study revealed that loss-of-function mutations of binding sites of B56, SMYD3 or VP30 in NP did not prevent the other two proteins colocalizing with NP-induced inclusions, indicating that these three binding sites functioned independently. When mapping binding sites of SMYD3 interacting with NP, we found that none of the internal deletion mutants in our study completely lost binding to NP. However, three mutants without N-SET, MYND or core-SET domain showed markedly impaired function in associating with NP, suggesting that the N-terminus of SMYD3 contributed to the interaction.

Depletion of SMYD3 significantly decreased EBOV mRNA, but not vRNA or cRNA. SMYD3 has been reported to interact with viral proteins of Human T-cell leukemia virus type 1 (HTLV-1), Hepatitis C virus (HCV) and Hepatitis B virus (HBV) [52–54]. In these reports, SMYD3 influenced viral protein-dependent signal transduction or virus particle production, but had no effect on viral RNA synthesis. For the first time, our study discovered that SMYD3 played a role in viral mRNA transcription. Further study showed that VP30-6A could partially rescue replication of EBOV after depletion of SMYD3. In addition, SMYD3 promoted VP30 binding to NP in a dose-dependent manner, indicating that SMYD3 acted as a positive regulator of NP-VP30 interaction to support viral transcription.

In summary, we identified SMYD3 as a novel host factor which was required for efficient replication of EBOV. SMYD3 associated with EBOV polymerase complex via specific interaction with NP and supported viral mRNA transcription through promoting NP-VP30 interaction.

## Supplementary Material

Supplemental Material
