# Supplementary figures and images for: Host factor SMYD3 is recruited by Ebola virus nucleoprotein to facilitate viral mRNA transcription
Source: Emerg Microbes Infect. 2019 Sep 13;8(1):1347–60. doi: 10.1080/22221751.2019.1662736 (PMC6758638; doi:10.1080/22221751.2019.1662736)

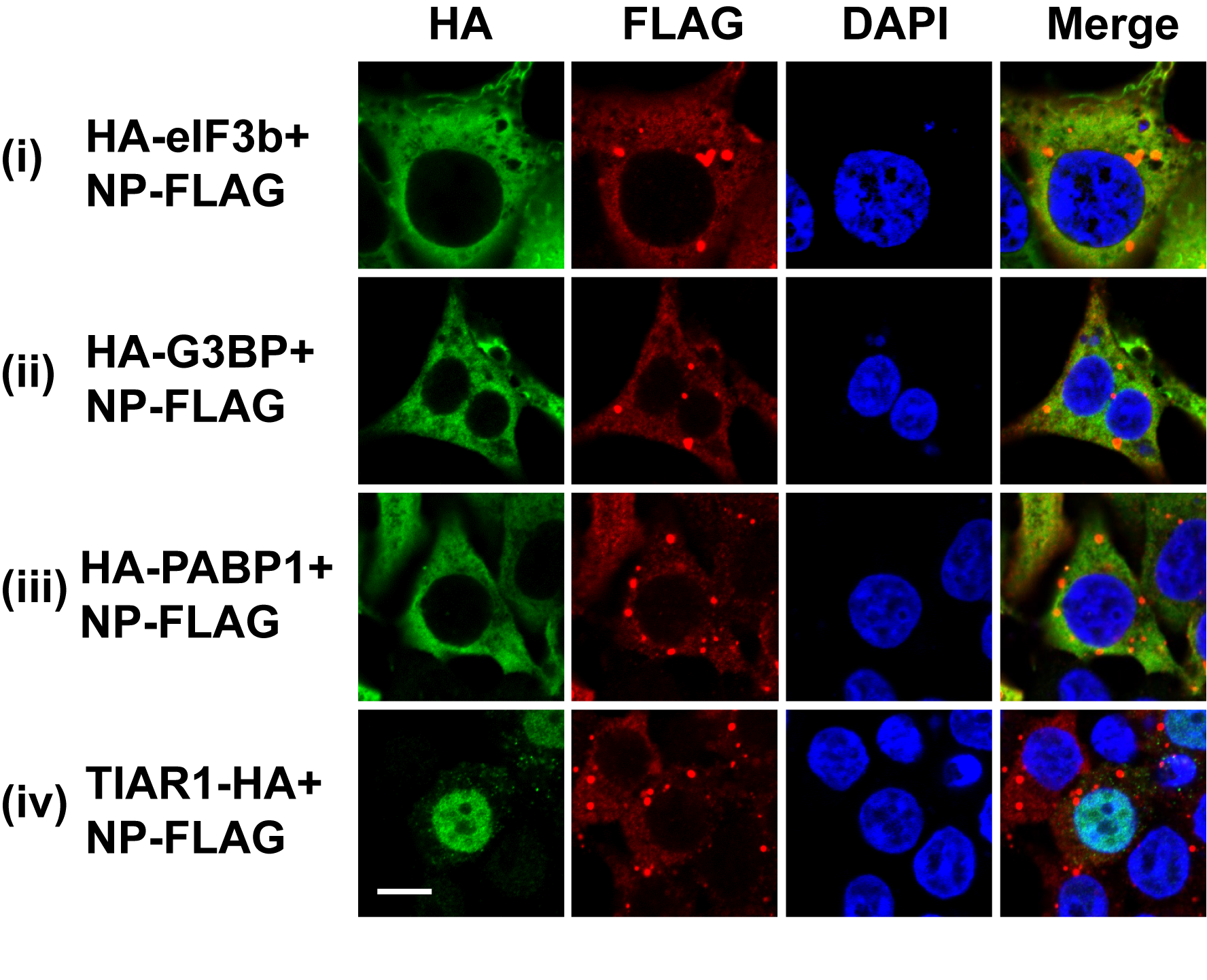

Supplement: Supplemental Material [file TEMI_A_1662736_SM3896.zip › supplementary figure 1_final.tif]
